# Acute Post‐Partum Psychosis and Dystonia Reveals Late‐Onset MPAN

**DOI:** 10.1002/mdc3.70774

**Published:** 2026-08-16

**Authors:** Mathilde Lachaume, Audrey Schalk, Nadège Calmels, Christine Tranchant, Ozanne de Andrade, Fabrice Berna, Mathieu Anheim, Thomas Wirth

**Affiliations:** ^1^ Service de Neurologie Hôpitaux Universitaires de Strasbourg Strasbourg France; ^2^ Laboratoire de diagnostic génétique Hôpitaux Universitaires de Strasbourg Strasbourg France; ^3^ Département de Médecine Translationnelle et Neurogénétique Institut de Génétique et de Biologie Moléculaire et Cellulaire, INSERM‐U964/CNRS‐UMR7104/Université de Strasbourg Illkirch‐Graffenstaden France; ^4^ Institut des Neurosciences de Strasbourg Université de Strasbourg Strasbourg France; ^5^ Service de psychiatrie Hôpitaux Universitaires de Strasbourg Strasbourg France

**Keywords:** dystonia‐parkinsonism, Electroconvulsivotherapy, genetics, mitochondrial membrane protein associated neurodegeneration, neurodegeneration with brain iron accumulation, post‐partum psychosis

Mitochondrial membrane protein associated neurodegeneration (MPAN) is a neurodegeneration with brain iron accumulation (NBIA), a rare group of neurogenetic diseases characterized by intracerebral iron deposits.^1^ It is caused by pathogenic variants in *C19orf12*, typically autosomal recessive, though autosomal dominant cases occur (mostly involving the last exon).^2^ Typical MPAN onset occurs in childhood (around age 10), manifesting with spastic paresis, progressive parkinsonism, dystonia, cognitive decline and variable neuropsychiatric disorders.^3^ We report a variant case with later, acute post‐partum onset and dominated by psychosis and dystonia related to the homozygous [NM_001031726.3]:c.32C>T; p.(Thr11Met) variant in *C19orf12*.

## Case Report

A 31‐year‐old Middle‐Eastern female presented with abrupt‐onset puerperal psychosis and catatonia following childbirth. Born to non‐consanguineous parents, after an unremarkable pregnancy, she had no prior medical history and no prior symptoms reported by family or documented by clinicians.

Two days postpartum (fourth delivery), she experienced acoustico‐verbal hallucinations, lead‐pipe rigidity and catatonia, associated with fever and sweating. Her condition worsened, with feeding and drinking difficulties, stupor, and catalepsy. Months later, after starting amisulpride, she developed trismus and dysphagia, complicated by aspiration pneumonia requiring intensive care. At discharge, she remained bedridden, with language alteration, although formal neuropsychological assessment was precluded by poor cooperation. She later experienced a single generalized tonic–clonic seizure, in context of benzodiazepine withdrawal.

Brain MRI was initially interpreted as normal. Extensive workup, including large biological investigation, CSF analysis, electroencephalogram, whole‐body CT and PET scan, was non‐contributory. A putative autoimmune encephalitis was eventually suspected but corticosteroids, plasma exchange and immunoglobulin infusions initiation had no effect.

One year post‐onset, the patient remained mutic and akinetic. Oculomotor tracking was preserved. Examination revealed fixed lower‐limb dystonia, diffuse hyporeflexia, indifferent plantar responses and bilateral Hoffman signs with upper limb rigidity (Video 1).

**Video 1:** MPAN clinical exam: video showing the clinical manifestation identified in the subject. It initially demonstrates upper limb myoclonus and right‐predominant dystonia, followed by bilateral Hoffmann's signs. Clinical examination reveals lead‐pipe rigidity and brisk deep tendon reflexes. In the lower limbs, dystonia is also present, characterized by an equinovarus posture. Patellar reflexes are brisk, and the Babinski sign is equivocal on the right.

**Figure 1:**
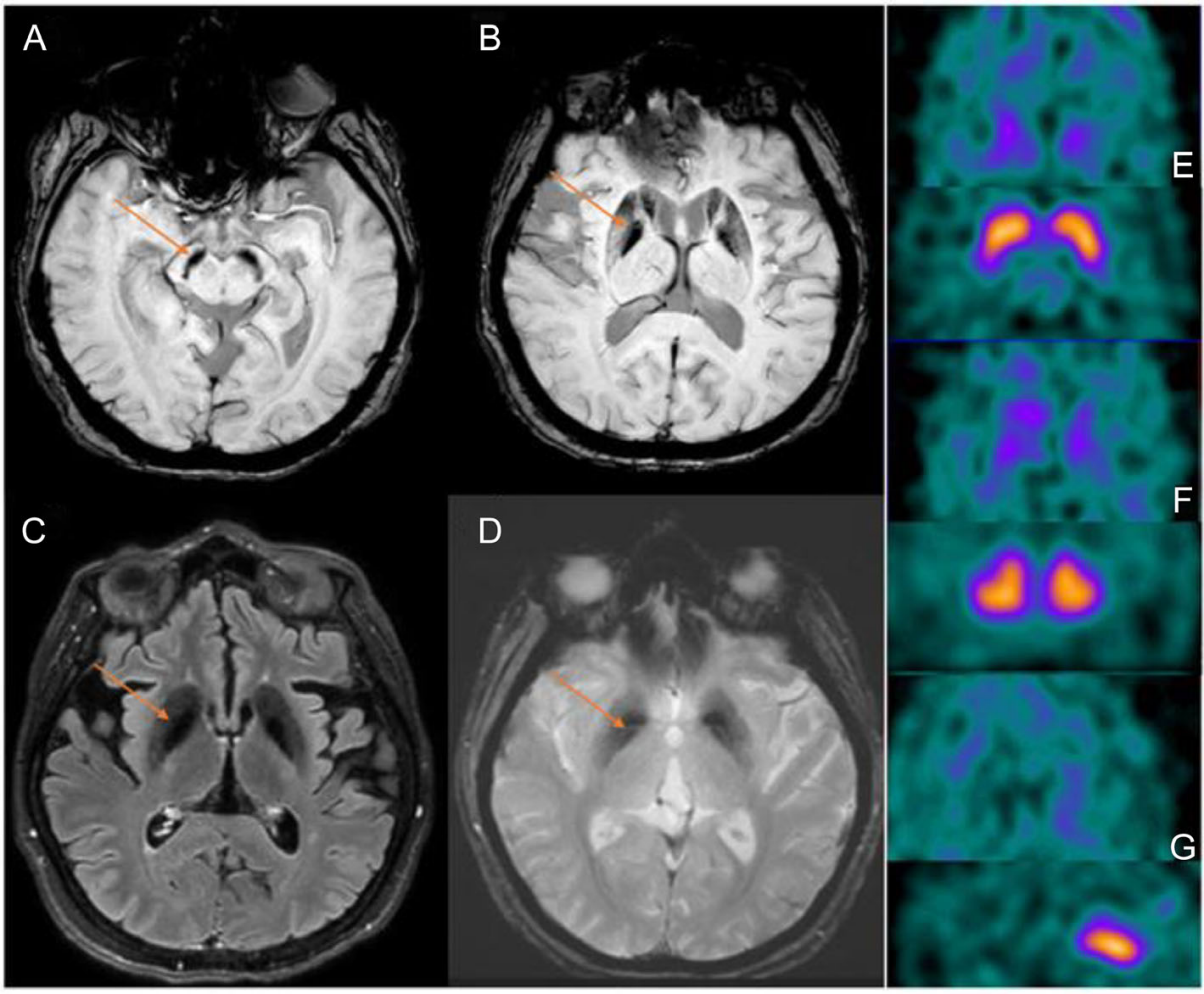
Axial magnetic resonance imaging shows bilateral hypointensity of the substantia nigra (orange arrow on a) and globus pallidus (orange arrow on b and c) on SWI (A, B) and Flair (C); and isointense linear signal of the medial medullary lamina on T2*(orange arrow on d). Ioflupane scintigraphy shows severe bilateral dopaminergic denervation (e. coronal f. axial g. sagittal; each image being followed just below by the normal image of a control case).

Brain MRI reanalysis revealed SWI hypointensities in the globus pallidus and substantia nigra and to a lesser extent, in the putamen and caudate nucleus, suggesting iron deposition. T2* sequences showed an isosignal of the medullary lamina interna, between the internal and external pallidi, characteristic of MPAN (Figure 1). Ioflupane scintigraphy highlighted severe bilateral nigro‐striatal dopaminergic denervation (Figure 1). Targeted gene panel sequencing revealed homozygous Hg19 chr19‐30,199,322 G>A C19ORF12:c.32C>T p.Thr11Met NM_001031726.3 biallelic pathogenic variant in *C19orf12*, confirming the diagnosis of MPAN (Supplemental methods S1, Supplemental Table S1 in Data S1). No other class IV/V ACMG variants or relevant variants of unknown significance were found. Although mimicking postpartum psychosis initially, the clinical course shifted toward an organic etiology, as motor and neuroimaging abnormalities emerged. Neuroleptic malignant syndrome was ruled out since symptoms predated neuroleptics and CPK levels remained normal.

Electroconvulsive therapy was initiated to treat catatony, and led to a partial improvement of symptoms. Levodopa therapy was also introduced but did not lead to a dramatic improvement of the motor phenomenology. Forty two months after symptoms onset she remained unable to walk independently, with a major neurocognitive disorders and language impairment although her psychiatric symptoms had significantly improved.

## Discussion

MPAN usually occurs around the age of 10, with a progressive onset, and leading to loss of walking in the majority of cases before the end of the second decade.^3^ Here we report an atypical case with: (i) abrupt onset; (ii) after 30 years of age; (iii) following pregnancy; and (iv) revealed by psychiatric symptoms. The clinical characteristics of this rare genetically determined neurodegenerative disease include psychiatric disorders, alteration of the upper motor neuron (spasticity, Babinski sign) as well as the lower motor neuron (peripheral motor neurogenic syndrome), cognitive deterioration and, most frequently, dystonia and parkinsonism, which was compatible with our patient's clinical presentation.^3^, ^4^ Progression is usually gradual, but cases of rapid onset and worsening have been reported. In the literature, the homozygous variant [NM_001031726.3]:c.32C>T; p.Thr11Met was associated with later symptoms onset, usually beginning in the third decade. A more severe and rapid progression was also described with this variant, similar to the one observed in our patient.^4^, ^5^ The Ioflupane (DaTscan®) scintigraphy reveals severe denervation, which likely predated delivery and may remain asymptomatic for several years, as described in other neurodegenerative conditions.^6^ To date, there are no documented cases of MPAN diagnosed following postpartum psychosis; however, it is known that pregnancy can trigger rapid and severe clinical deterioration, including the worsening of psychiatric symptoms. Hormonal and metabolic shifts during pregnancy (notably the disruption of coenzyme A metabolism) could accelerate disease progression.^7^ Age may also have been a sensitizing factor in our patient, eroding her brain resilience within the context of latent MPAN, leading to postpartum decompensation, as previously documented in this disorder.^2^

This case also emphasizes the critical importance of early imaging review by a multidisciplinary team in cases of atypical postpartum psychosis. In this instance, the initial hypothesis of autoimmune encephalitis induced a confirmation bias during imaging interpretation, leading to a diagnostic delay that was only corrected once a neurodegenerative etiology was finally considered.^8^

There is currently no consensus on therapeutic management.^9^, ^10^ Dopaminergic treatment may have a beneficial effect on parkinsonian syndrome, but only minimally improved the parkinsonism in our patient. The use of electroconvulsive therapy, which showed some benefit in our patient, was not previously described in the literature to our knowledge.

To conclude, our work enriches clinical knowledge of MPAN by demonstrating that rare neurogenetic conditions can sometimes brutally occur and mimic non‐genetic conditions such as autoimmune encephalitis including severe psychiatric disturbances. There is currently no disease‐modifying treatment for MPAN, but electro convulsive therapy appears to be an interesting avenue for improving psychiatric disorder of this severe and debilitating disorder.

## Author Roles

Research project: (1) A. Conception, B. Design, C. Acquisition of data, D. Analysis and interpretation of data; (2) Manuscript: A. Writing of the first draft, B. Review and critique; (3) Other: A. Subject recruitment, B. Clinical assessment of patients, C. Study supervision.

M.L.: 1B, 1C, 1D, 2A, 3A, 3B.

A.S.: 1C, 1D, 2B.

N.C.: 1C, 1D, 2B.

C.T.: 1C, 1D, 2B.

F.B.: 1C, 1D, 2B.

M.A.: 1C, 1D, 2B, 3C.

T.W.: 1A, 1B, AC, 1D, 2B, 3A, 3B, 3C.

## Disclosures

**Ethical Compliance Statement:** As the patient lacked the capacity to provide consent, written and verbal informed consent was obtained from their legally authorized representative for the clinical description and the accompanying video. Written informed consent for video recording and publication was approved by Strasbourg University Hospital local ethics committee. The authors have read and comply with the Journal's ethical publication guidelines.

**Funding Sources and Conflict of Interest:** No specific funding was used for this work.

**Financial Disclosures for the Previous 12 Months:** M.A. reports grants from IPSEN, Abbvie, Orkyn, Teva, Reata Pharmaceuticals, Biogen, Aguettant, Merz and Ever Pharma, consulting fees from IPSEN, Abbvie, Orkyn, Teva, Reata Pharmaceuticals, Biogen, Aguettant, Merz and Ever Pharma, payment or honoraria for lectures, presentations, speakers bureaus, manuscript writing or educational events from IPSEN, Abbvie, Orkyn, Teva, Reata Pharmaceuticals, Biogen, Aguettant, Merz and Ever Pharma, payment for expert testimony from Biogen, support for attending meetings and/or travel from IPSEN, Abbvie, Orkyn, Teva, Reata Pharmaceuticals, Biogen, Aguettant, Merz and Ever Pharma and participation on a Data Safety Monitoring Board or Advisory Board for Abbvie and Biogen. T.W. reports grants from Direction Générale de l’Offre de soins, France Parkinson, and Association Connaitre les Syndromes Cérébelleux, payment or honoraria for lectures, presentations, speakers bureaus, manuscript writing or educational events from Abbvie, Ipsen and Biogen, payment for expert testimony from Abbvie, Support for attending meetings and/or travel from Homeperf, Lundbeck and The Movement Disorders Society. The rest of the authors declare no conflict of interest.

## Ethics Statement

As the patient lacked the capacity to provide consent, written and verbal informed consent was obtained from their legally authorized representative for the clinical description and the accompanying video. Written informed consent for video recording and publication was approved by Strasbourg University Hospital local ethics committee. The authors have read and comply with the Journal's ethical publication guidelines.

## Supporting information


**Data S1.** Supplemental methods.

## Data Availability

The data that support the findings of this study are available on request from the corresponding author. The data are not publicly available due to privacy or ethical restrictions.
