# Supplementary material for: Acute Post‐Partum Psychosis and Dystonia Reveals Late‐Onset MPAN
Source: Mov Disord Clin Pract. 2026 Aug 16:10.1002/mdc3.70774. Online ahead of print. doi: 10.1002/mdc3.70774 (PMC13477866; doi:10.1002/mdc3.70774)
Supplement: Supplementary file 1 [file MDC3-9999-0-s001.pdf]

Supplementary Methods

Mathilde Lachaume MD; Audrey Schalk MD; Nadège Calmels PharmD, PhD; Christine Tranchant MD, PhD; Ozanne de Andrade MD ;Fabrice Berna MD, PhD; Mathieu Anheim MD, PhD; Thomas Wirth MD, PhD

eMethods: Genetic supplemental methods

eTable 1.: Name, phenotype and inheritance of the genes included in the panel. (Montaut S. et al[1])

We manually processed each series DNA samples corresponding to index case for library preparation, and then libraries were pooled for capture and enrichment reaction. For the sequencing step, we used the Nextseq550 Sequencing System (Illumina) flow cell for the targeted sequencing of DNA samples. The final step corresponded to the bioinformatics analysis of sequencing data, the interpretation of the variants, and the writing of diagnostic reports. The genes included in the panel are listed in eTable 1 below. Analysis included identification of variants of unknown significance (VUSs). Novel variants were considered as VUSs when they were rare (minor allele frequency < 0.1%) in population databases (eg, Exome Variant Server, Exome Aggregation Consortium, gnomAD, and 1000 Genomes Browser) and/or predicted to be pathogenic by prediction tools (eg, Sorting Intolerant From Tolerant [SIFT], PolyPhen, and MutationTaster), but when the phenotype expressed by the patient was not consistent with the usual phenotype described in the literature. Copy Number Variant calling was perfomed using CANOES[2].

Bibliography:

1. Montaut, S. *et al.* Assessment of a Targeted Gene Panel for Identification of Genes Associated With Movement Disorders. *JAMA Neurol.* **75**, 1234 (2018).
2. Backenroth, D. *et al.* CANOES: detecting rare copy number variants from whole exome sequencing data. *Nucleic Acids Res.* **42**, e97 (2014).

eTable 1. Name, phenotype and inheritance of the genes included in the panel.

Name	refSeq	Disease	Phenotype MIM Number	Inheritance
SNCA including Rep1 promoter region	NM_001146055	Dementia, Lewy body	127750	AD
		Parkinson disease 1	168601	AD
		Parkinson disease 4	605543	AD
LRRK2	NM_198578	{Parkinson disease 8}	607060	AD
GBA	NM_001005742	Gaucher disease	608013, 230800, 230900, 231000, 231005	AR
		{Lewy body dementia, susceptibility to}	127750	AD
		{Parkinson disease, late-onset, susceptibility to}	168600	AD
PINK1	NM_032409	Parkinson disease 6, early onset	605909	AR
DJ-1/PARK7	NM_007262	Parkinson disease 7, autosomal recessive early-onset	606324	AR
UCHL1	NM_004181	?Neurodegeneration with optic atrophy, childhood onset	615491	AR
		?{Parkinson disease 5, susceptibility to}	613643	AD
GIGYF2	NM_001103146	{Parkinson disease 11}	607688	AD
parkin/PARK2	NM_004562	Parkinson disease, juvenile, type 2	600116	AR
HTRA2	NM_013247	3-methylglutaconic aciduria, type VIII	617248	AR
		{Parkinson disease 13}	610297	AD
VPS35	NM_018206	{Parkinson disease 17}	614203	AD
EIF4G1	NM_001194947	{Parkinson disease 18}	614251	AD
DNAJC6	NM_001256864	Parkinson disease 19a, juvenile-onset	615528	AR
		Parkinson disease 19b, early-onset	615528	AR
DNAJC13	NM_015268	Reclassified - Variant of unknown significance Parkinson disease 21 - AD		AD
SYNJ1/synaptojanin 1	NM_003895	Parkinson disease 20, early-onset	615530	AR
RAB7L1	NM_001135662	{Parkinson disease 16}	613164	AD
PANK2	NM_153638	HARP syndrome	607236	AR
		Neurodegeneration with brain iron accumulation 1	234200	AR
PLA2G6	NM_003560	Infantile neuroaxonal dystrophy 1	256600	AR
		Neurodegeneration with brain iron accumulation 2B	610217	AR
		Parkinson disease 14, autosomal recessive	612953	AR
ATP13A2	NM_022089	?Ceroid lipofuscinosis, neuronal, 12	606693	AR
		Kufor-Rakeb syndrome	606693	AR
SLC6A3	NM_001044	Parkinsonism-dystonia, infantile	613135	AR
FBXO7	NM_012179	Parkinson disease 15, autosomal recessive	260300	AR
SPG11 / spatacsin	NM_025137	Amyotrophic lateral sclerosis 5, juvenile	602099	AR
		Spastic paraplegia 11, autosomal recessive	604360	AR
SPG15 / ZFYVE26	NM_015346.3	Spastic paraplegia 15, autosomal recessive	270700	AR
POLG / POLG1	NM_001126131	Mitochondrial DNA depletion syndrome 4A (Alpers type)	203700	AR
		Mitochondrial DNA depletion syndrome 4B (MNGIE type)	613662	AR
		Mitochondrial recessive ataxia syndrome (includes SANDO and SCAE)	607459	AR
MAPT	NM_001123066	Dementia, frontotemporal, with or without parkinsonism	600274	AD
		Supranuclear palsy, progressive	601104	AD
		Supranuclear palsy, progressive atypical	260540	AR
		{Parkinson disease, susceptibility to}	168600	AD
GRN / progranulin	NM_002087	Ceroid lipofuscinosis, neuronal, 11	614706	AR
		Frontotemporal lobar degeneration with ubiquitin-positive inclusions	607485	AD
DCTN1 / dynactin 1	NM_004082	Perry syndrome	168605	AD

© 2018 American Medical Association. All rights reserved.

Continued

eTable 1. Continued

Name	refSeq	Disease	Phenotype MIM Number	Inheritance
ATP6AP2	NM_005765	?Parkinsonism with spasticity, X-linked	300911	AR
HTT / huntingtin	NM_002111	Huntington disease	143100	AD
JPH1 / junctophilin 1	NM_020647	Huntington-disease like	NR	NR
VPS13A	NM_033305	Choreoacanthocytosis	200150	AR
XK	NM_021083	McLeod syndrome with or without chronic granulomatous disease	300842	XL
NKX2-1 / TITF1	NM_001079668	Chorea, hereditary benign	118700	AD
		Choreoathetosis, hypothyroidism, and neonatal respiratory distress	610978	AD
ATP7B	NM_000053	Wilson disease	277900	AR
FTL	NM_000146	Neurodegeneration with brain iron accumulation 3	606159	AD
FA2H	NM_024306	Spastic paraplegia 35, autosomal recessive	612319	AR
WDR45	NM_001029896	Neurodegeneration with brain iron accumulation 5	300894	XLD
CP / ceruleoplasmin	NM_000096	Cerebellar ataxia	604290	AR
		Hemosiderosis, systemic, due to aceruloplasminemia	604290	AR
		[Hypoceruloplasminemia, hereditary]	604290	AR
C19orf12	NM_001031726	?Spastic paraplegia 43, autosomal recessive	615043	AR
		Neurodegeneration with brain iron accumulation 4	614298	AR
DCAF17 / C2orf37	NM_025000	Woodhouse-Sakati syndrome	241080	AR
COASY	NM_001042532	Neurodegeneration with brain iron accumulation 6	615643	AR
PRNP / prion protein	NM_001271561	Creutzfeldt-Jakob disease	123400	AD
		Gerstmann-Straussler disease	137440	AD
		Huntington disease-like 1	603218	AD
TPP1 / CLN2	NM_000391	Ceroid lipofuscinosis, neuronal, 2	204500	AR
		Spinocerebellar ataxia, autosomal recessive 7	609270	AR
CLN3	NM_001042432	Ceroid lipofuscinosis, neuronal, 3	204200	AR
HEXA	NM_000520	GM2-gangliosidosis, several forms	272800	AR
		Tay-Sachs disease	272800	AR
HEXB	NM_000521	Sandhoff disease, infantile, juvenile, and adult forms	268800	AR
GCH1	NM_000161	Dystonia, DOPA-responsive, with or without hyperphenylalaninemia	128230	AD, AR
TH	NM_199292	Segawa syndrome, recessive	605407	AR
SPR	NM_003124	Dystonia, dopa-responsive, due to sepiapterin reductase deficiency	612716	?AD, AR
NPC1	NM_000271	Niemann-Pick disease, type C1 and D	257220	AR
NPC2	NM_006432	Niemann-pick disease, type C2	607625	AR
TIMM8A / DDP	NM_004085	Mohr-Tranebjaerg syndrome	304700	XLR
SUCLA2	NM_003850	Mitochondrial DNA depletion syndrome 5 (encephalomyopathic with or without methylmalonic aciduria)	612073	AR
GM2A	NM_000405	GM2-gangliosidosis, AB variant	272750	AR
GLB1	NM_000404	GM1-gangliosidosis, type I, II and III	230500, 230600, 230650	AR
		Mucopolysaccharidosis type IVB (Morquio)	253010	AR
TOR1A / torsin 1A	NM_000113	Dystonia-1, torsion	128100	AD
TAF1	NM_004606	Dystonia-Parkinsonism, X-linked	314250	XLR
TUBB4A / tubulin beta 4A	NM_006087	Dystonia 4, torsion, autosomal dominant	128101	AD
		Leukodystrophy, hypomyelinating, 6	612438	AD
SGCE / epsilon sarcoglycan	NM_001099400	Dystonia-11, myoclonic	159900	AD
THAP1	NM_018105	Dystonia 6, torsion	602629	AD
PRKRA	NM_003690	Dystonia 16	612067	AR
SLC6A3 / DAT	NM_001044	Parkinsonism-dystonia, infantile	613135	AR
CIZ1	NM_001257975	?Dystonia 23	614860	AD

© 2018 American Medical Association. All rights reserved.

Continued

eTable 1. Continued

Name	refSeq	Disease	Phenotype MIM Number	Inheritance
ANO3 / anoctamin 3	NM_031418	Dystonia 24	615034	AD
GNAL	NM_182978	Dystonia 25	615073	AD
ATM	NM_000051	Ataxia-telangiectasia	208900	AR
HPRT1	NM_000194	Lesch-Nyhan syndrome	300322	XLR
GCDH	NM_000159	Glutaricaciduria, type I	231670	AR
FOXRED1, NDUFA2, NDUFA9, NDUFA10, NDUFA12, NDUFAF2, NDUFAF6/c8orf38, NDUFS3, NDUFS4, NDUFS7, NDUFS8	NM_017547, NM_002488, NM_005002, NM_004544, NM_018838, NM_174889, NM_152416, NM_004551, NM_002495, NM_024407, NM_002496	Leigh syndrome due to mitochondrial complex I deficiency	256000	Mi, AR, XL
SDHA	NM_004168	Leigh syndrome	256000	Mi, AR
		Mitochondrial respiratory chain complex II deficiency	252011	AR
DLD	NM_000108	Dihydrolipoamide dehydrogenase deficiency	246900	AR
SURF1 / surfeit 1	NM_003172	Leigh syndrome, due to COX IV deficiency	256000	Mi, AR
COX15	NM_078470	Leigh syndrome due to cytochrome c oxidase deficiency	256000	Mi, AR
SLC19A3	NM_025243	Thiamine metabolism dysfunction syndrome 2 (biotin- or thiamine-responsive encephalopathy type 2)	607483	AR
DRD5	NM_000798	Dystonia, primary cervical		
		{Blepharospasm, primary benign}	606798	IC
ACTB / beta actin	NM_001101	?Dystonia, juvenile-onset	607371	AD
ETHE1	NM_014297	Ethylmalonic encephalopathy	602473	AR
COL4A1	NM_001845	Angiopathy, hereditary, with nephropathy, aneurysms, and muscle cramps	611773	AD
		Brain small vessel disease with or without ocular anomalies	607595	AD
		Porencephaly 1	175780	AD
FUCA1 / alpha L fucosidase	NM_000147	Fucosidosis	230000	AR
GAMT	NM_138924	Cerebral creatine deficiency syndrome 2	612736	AR
SCP2	NM_002979	Leukoencephalopathy with dystonia and motor neuropathy	613724	AR
AP4B1	NM_001253852	Spastic paraplegia 47, autosomal recessive	614066	AR
PRKCG	NM_002739	Spinocerebellar ataxia 14	605361	AD
ALDH5A1	NM_170740	Succinic semialdehyde dehydrogenase deficiency	271980	AR
SUOX	NM_001032387	Sulfite oxidase deficiency	272300	AR
APTX / aprataxin	NM_001195248	Ataxia, early-onset, with oculomotor apraxia and hypoalbuminemia	208920	AR
SETX / senataxin	NM_015046	Spinocerebellar ataxia, autosomal recessive 1	606002	AR
ARSA / arylsulfatase A	NM_001085425	Metachromatic leukodystrophy	250100	AR
GALC / galactosylceramidase	NM_000153	Krabbe disease	245200	AR
ABCD1	NM_000033	Adrenoleukodystrophy, adult adrenomyeloneuropathy	300100	XLR
MMADHC / c2orf25	NM_015702	Methylmalonic aciduria and homocystinuria, cblD type	277410	AR
SERAC1	NM_032861	3-methylglutaconic aciduria with deafness, encephalopathy, and Leigh-like syndrome	614739	AR
SDHAF1	NM_001042631	Mitochondrial complex II deficiency	252011	AR
ARX	NM_139058	Partington syndrome	309510	XLR
PLP1	NM_000533	Pelizaeus-Merzbacher disease	312080	XLR
		Spastic paraplegia 2, X-linked	312920	XLR

© 2018 American Medical Association. All rights reserved.

Continued

eTable 1. Continued

Name	refSeq	Disease	Phenotype MIM Number	Inheritance
SEPSECS	NM_016955	Pontocerebellar hypoplasia type 2D	613811	AR
ALDH7A1	NM_001201377	Epilepsy, pyridoxine-dependent	266100	AR
MECP2	NM_001110792	Rett syndrome	312750	XLD
PRRT2	NM_001256442	Convulsions, familial infantile, with paroxysmal choreoathetosis	602066	AD
		Episodic kinesigenic dyskinesia 1	128200	AD
SLC2A1 / GLUT1	NM_006516	Dystonia 9	601042	AD
		GLUT1 deficiency syndrome 1, infantile onset, severe	606777	AD, AR
		GLUT1 deficiency syndrome 2, childhood onset	612126	AD
PNKD / MR1 / DYT8	NM_015488	Paroxysmal nonkinesigenic dyskinesia	118800	AD
ATP1A3	NM_152296	Alternating hemiplegia of childhood 2	614820	AD
		CAPOS syndrome	601338	AD
		Dystonia-12	128235	AD
KCNMA1	NM_001161352	Generalized epilepsy and paroxysmal dyskinesia	609446	AD
NOL3	NM_001185057	Myoclonus, familial cortical	614937	AD
ADCY5	NM_183357	Dyskinesia, familial, with facial myokymia	606703	AD
SLC16A2 / MCT8	NM_006517	Allan-Herndon-Dudley syndrome	300523	XLD
CACNA1A	NM_023035	Episodic ataxia, type 2	108500	AD
		Migraine, familial hemiplegic, 1, with or without progressive cerebellar ataxia	141500	AD
		Spinocerebellar ataxia 6	183086	AD
KCNA1	NM_000217	Episodic ataxia/myokymia syndrome	160120	AD
CACNB4	NM_000726	Episodic ataxia, type 5	613855	AD
SLC1A3	NM_004172	Episodic ataxia, type 6	612656	AD
PDHX	NM_003477	Lacticacidemia due to PDX1 deficiency	245349	AR
PDHA1	NM_001173454	Pyruvate dehydrogenase E1-alpha deficiency	312170	XLD
GLRA1	NM_001146040	Hyperekplexia, hereditary 1, autosomal dominant or recessive	149400	AD, AR
GLRB	NM_000824	Hyperekplexia 2, autosomal recessive	614619	AR
COMT	NM_000754	COMT-activity polymorphism	NR	NR
SLC30A10	NM_018713	Hypermanganesemia with dystonia 1	613280	AR
FOXG1	NM_005249	Rett syndrome, congenital variant	613454	IC
RAB39B	NM_171998	?Waisman syndrome	311510	XLR

AD: autosomal dominant, AR: autosomal recessive, XLD: X-linked dominant, XLR: X-linked recessive, Mi: mitochondrial, {}: incomplete penetrance, ?: debated, NR: not reported.

© 2018 American Medical Association. All rights reserved.
